# Hepatitis B virus X protein (HBx)-induced abnormalities of nucleic acid metabolism revealed by ^1^H-NMR-based metabonomics

**DOI:** 10.1038/srep24430

**Published:** 2016-04-14

**Authors:** Dan Yue, Yuwei Zhang, Liuliu Cheng, Jinhu Ma, Yufeng Xi, Liping Yang, Chao Su, Bin Shao, Anliang Huang, Rong Xiang, Ping Cheng

**Affiliations:** 1 State Key Laboratory of Biotherapy and Cancer Center/Collaborative Innovation Center for Biotherapy, West China Hospital, Sichuan University, Chengdu 610041, PR China; 2 Division of Endocrinology and Metabolism, West China Hospital of Sichuan University, Chengdu, China; 3 Oncology Medicine Department, Donggang Branch of The First Hospital of Lanzhou University, 730000, PR China; 4 Department of Pathology, West China Second University Hospital, Sichuan University, Chengdu 610041, PR China; 5 School of Medicine/Collaborative Innovation Center for Biotherapy, Nankai University, Tianjin 300071, PR China

## Abstract

Hepatitis B virus X protein (HBx) plays an important role in HBV-related hepatocarcinogenesis; however, mechanisms underlying HBx-mediated carcinogenesis remain unclear. In this study, an NMR-based metabolomics approach was applied to systematically investigate the effects of HBx on cell metabolism. EdU incorporation assay was conducted to examine the effects of HBx on DNA synthesis, an important feature of nucleic acid metabolism. The results revealed that HBx disrupted metabolism of glucose, lipids, and amino acids, especially nucleic acids. To understand the potential mechanism of HBx-induced abnormalities of nucleic acid metabolism, gene expression profiles of HepG2 cells expressing HBx were investigated. The results showed that 29 genes involved in DNA damage and DNA repair were differentially expressed in HBx-expressing HepG2 cells. HBx-induced DNA damage was further demonstrated by karyotyping, comet assay, Western blotting, immunofluorescence and immunohistochemistry analyses. Many studies have previously reported that DNA damage can induce abnormalities of nucleic acid metabolism. Thus, our results implied that HBx initially induces DNA damage, and then disrupts nucleic acid metabolism, which in turn blocks DNA repair and induces the occurrence of hepatocellular carcinoma (HCC). These findings further contribute to our understanding of the occurrence of HCC.

With a high degree of malignancy, rapid progression, and high mortality rate, hepatocellular carcinoma (HCC) is the third most frequent cause of cancer-related death worldwide^1^. Hepatitis B virus (HBV) infection is a major risk factor for HCC^2^. In recent years, ~2 billion individuals have been found to be infected with HBV, of which, more than 350 million are chronically infected^3^,^4^. Thus, HBV has become one of the most important pathogens threatening human health.

As is well known, the main function of the liver is metabolism, thus plays a central role in metabolism of glucose, lipids, amino acids and nucleic acids. HBV infection leads to liver dysfunction, which in turn induces alterations of metabolism. Several recent studies have shown that HBV-related liver disease resulted in many metabolic abnormalities. One study showed that metabolite components of HCC markedly differed from those of adjacent non-tumor tissues, and low-grade HCC had clear metabolomics differences from high-grade HCC^5^. Another study reported that HBV-related HCC exhibited glucose and lipid metabolic abnormalities^6^. The levels of many indicators associated with lipid metabolism are altered in patients with chronic HBV infection^7^,^8^. Additionally, HBV infection can induce other abnormalities of metabolism such as amino acids, cholesterol, and choline^9^,^10^,^11^.

Hepatitis B virus X protein (HBx) is a multifunctional regulator, which is involved in viral replication, transcriptional regulation, cell cycle progression, DNA repair, apoptosis, and genetic stability^12^. Recently, several studies have also shown that HBx can disturb cell metabolism. HBx can interact with peroxisome proliferator-activated receptor-γ (PPAR-γ) and sterol regulatory element binding protein 1, increase their respective mRNA and protein expression, and lead to lipid accumulation in hepatocytes^7^. HBx can induce activation of lipogenic genes and fatty acid accumulation, which contributes to disease progression by promoting steatosis and liver inflammation in transgenic mice^13^. However, no systematic investigations about the effects of HBx on cell metabolism have been reported thus far.

Metabonomics is a systematic approach in studying low-molecular-weight compounds in cells, tissues and biofluids, which directly reflects the phenotypic features of an organism and provides important information on disease processes, biochemical functions, and drug toxicity^14^,^15^. Here, an NMR-based metabolomics approach was applied to study the mechanisms of HBx-mediated carcinogenesis. Studying the HBx-related mechanisms contributes to understanding of the occurrence of HCC.

## Results

### HBx affected the metabolic profiles of hepatic cells

**Figure 1:**
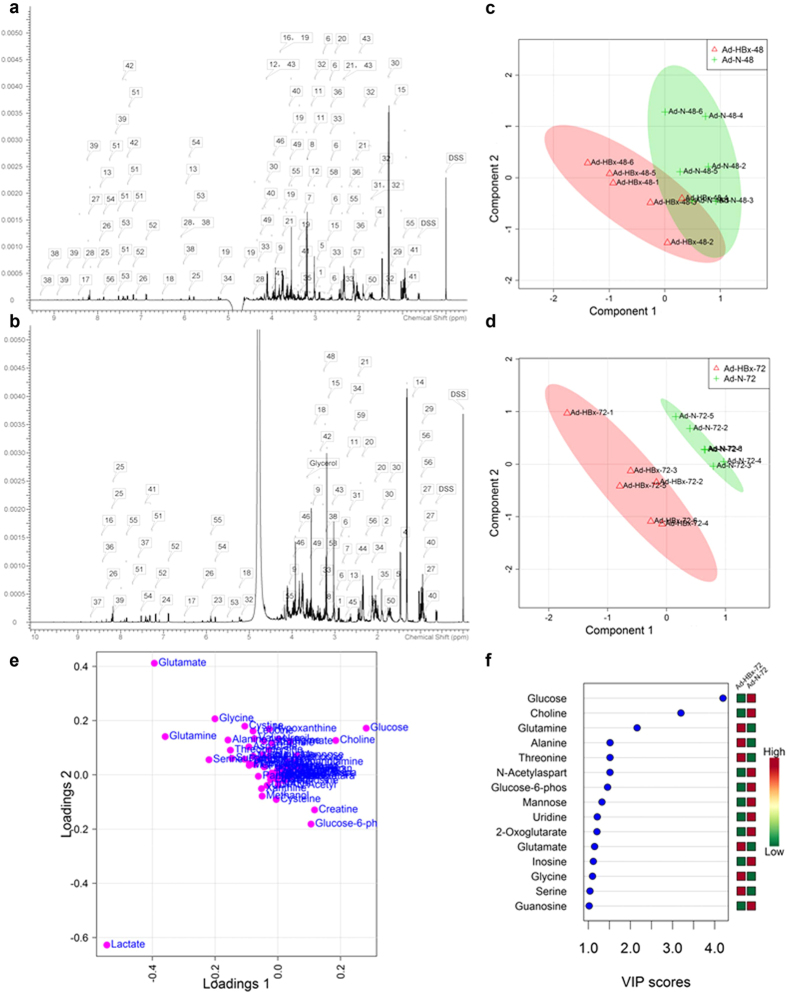
Representative 600-MHz ^1^H-NMR spectra and PLS-DA analysis of ^1^H-NMR spectral data. (**a**,**b**) Respectively show the spectra (0–10 ppm) of HepG2 cells infected by Ad-N and Ad-HBx, which included all metabolites identified. (**c**,**d**) Show the PLS-DA scores plots. (**c**) Ad-HBx-48 (red triangles) *versus* Ad-N-48 (green crosses) (R^2^ = 0.994 and Q^2^ = 0.447); (**d**) Ad-HBx-72 (red triangles) *versus* Ad-N-72 (green crosses) (R^2^ = 0.997 and Q^2^ = 0.743). The values of R^2^ and Q^2^ represent the goodness of fit and predictability of the models, respectively. (**e**) Loadings plot of 72 h post-infection from the PLS-DA analysis. (**f**) VIP scores of important metabolites. Red or green on the right indicates the low or high concentration of metabolites by comparing the concentration of each metabolite in Ad-HBx-72 and Ad-N-72.

To investigate the systematic effects of HBx on cell metabolism, high-resolution ^1^H-NMR spectroscopy was used. Fig. 1a,b showed the representative 600-MHz ^1^H-NMR spectra of HepG2 cells infected by control adenovirus (Ad-N) and HBx-expressing recombinant adenovirus (Ad-HBx), respectively. The spectra were similar to those of HepG2 cells infected by Ad-HBx, except for the different heights. Spectral resonances of metabolites were assigned according to the Chenomx Library. Sixty metabolites including nucleic acid components, amino acids and their derivatives, organic acids, sugars and amines were identified and quantified using Chenomx software based on the known concentration of 2, 2-dimethyl-2-silapentane-5-sulfonate (DSS) standard. Details of the metabolites are summarized in Table 1.

### HBx disrupted the metabolism of glucose, lipids and amino acids

PLS-DA, a multivariate statistical analysis, was performed on the NMR data to investigate the intrinsic differences in the metabolite levels between the groups at 48 h or 72 h post-infection. As shown in Fig. 1c, there was no distinct separation in scores plot of PLS-DA analysis between Ad-HBx-48 and Ad-N-48. However, Fig. 1d showed a clear separation between Ad-HBx-72 and Ad-N-72 with an R^2^ of 0.997 and a Q^2^ of 0.743.

The PLS-DA loadings plot can further identify spectral regions responsible for the separation observed in the scores plot. Fig. 1e showed loadings plot at 72 h post-infection, and points further from the center contributed most extensively to the variance. Metabolites such as glucose, choline, creatine, lactate, glutamate, glutamine, cysteine, cystine, acetate, serine, glycine and aspartate were important for the separation. The metabolites responsible for the classification of Ad-HBx-72 and Ad-N-72 were also identified using the variable importance in projection (VIP) scores. Metabolites with high VIP are more important in providing class separation, while those with small VIP provide less contribution^16^. The metabolites with VIP ≥ 1 were shown in Fig. 1f. Metabolites such as glucose, choline, glutamine, alanine, threonine, N-acetylaspartate and glucose-6-phosphate had higher VIP scores. Red or green on the right of Fig. 1f indicated the low or high concentration of metabolites by comparing the concentration of each metabolite in Ad-HBx-72 and Ad-N-72. The concentration of some metabolites such as glucose, choline and glucose-6-phosphate was reduced following Ad-HBx infection.

### HBx induced abnormalities of nucleic acid metabolism

**Figure 2:**
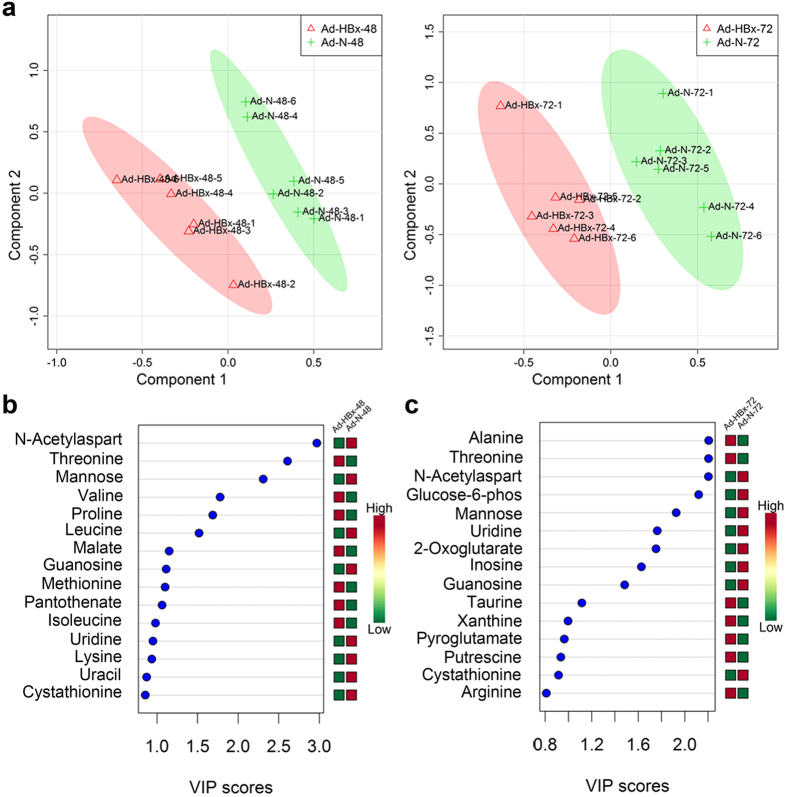
HBx-induced abnormalities of nucleic acids. (**a**) Scores plots of Ad-HBx-48 (red triangles) and Ad-N-48 (green crosses), and Ad-HBx-72 (red triangles) and Ad-N-72 (green crosses). (**b**) VIP scores of Ad-HBx-48 and Ad-N-48. (**c**) VIP scores of Ad-HBx-72 and Ad-N-72. Red or green on the right of (**b**) and (**c**) indicates the low or high concentration of metabolites by comparing the concentration of each metabolite in Ad-HBx-48 and Ad-N-48 or Ad-HBx-72 and Ad-N-72, respectively.

To further identify the metabolite biomarkers induced by HBx, we removed some metabolites and selected 45 components for PLS-DA analysis. As shown in Fig. 2a, the scores plots of Component 1 and Component 2 indicated that groups of Ad-HBx-48 and Ad-HBx-72 could be separated from Ad-N-48 and Ad-N-72, respectively. The parameters of the corresponding PLS-DA model were as follows: Ad-HBx-48 *versus* Ad-N-48: R^2^ = 0.998, Q^2^ = 0.608; Ad-HBx-72 *versus* Ad-N-72: R^2^ = 0.997, Q^2^ = 0.699. Figure 2b,c showed the higher VIP scores of identified metabolites. The concentration of nucleic acid components (data not shown) was relatively low, but uridine, inosine, guanosine, uracil and xanthine were important in providing class separation. As shown in Fig. 2b,c, at 48 h post-infection, the VIP scores of guanosine and uridine were ~1.1 and 1, respectively, and at 72 h post-infection, their scores were ~1.5 and 1.8, respectively. In addition, at 72 h post-infection, inosine and xanthine had higher VIP scores.

**Figure 3:**
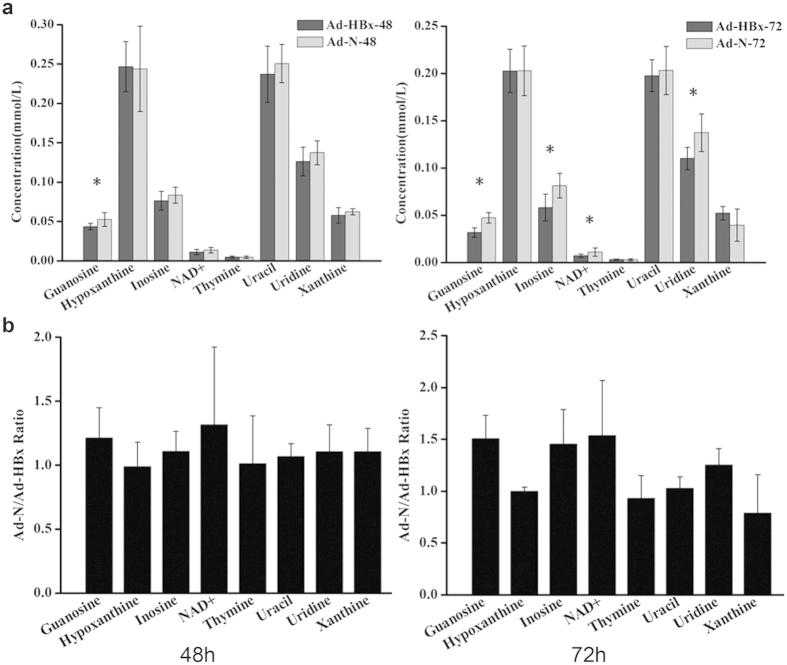
Quantification of nucleic acid components in HepG2 cells infected by Ad-HBx or Ad-N. (**a**) Comparison of nucleic acid components between Ad-HBx-48 and Ad-N-48 or Ad-HBx-72 and Ad-N-72 by Student’s *t* test. *indicates *p* < 0.05. (**b**) Ad-N to Ad-HBx ratio for each nucleic acid component identified at 48 h and 72 h after infection.

Student’s *t* test was applied to nucleic acid components identified by ^1^H-NMR. Figure 3 showed that at 48 h post-infection, guanosine had a significant decrease (*p* < 0.05), and its mean concentration of Ad-HBx-48 was 1.2-fold lower than Ad-N-48. At 72 h post-infection, several compounds in Ad-HBx-infected cells showed a significant decrease (*p* < 0.05), including guanosine (1.5-fold), inosine (1.5-fold), NAD^+^ (1.5-fold) and uridine (1.3-fold). Uridine, guanosine, inosine, uracil and xanthine are important intermediates of nucleic acid metabolism. Therefore, we speculated that HBx could induce nucleic acid metabolism abnormalities.

### HBx inhibited DNA synthesis

**Figure 4:**
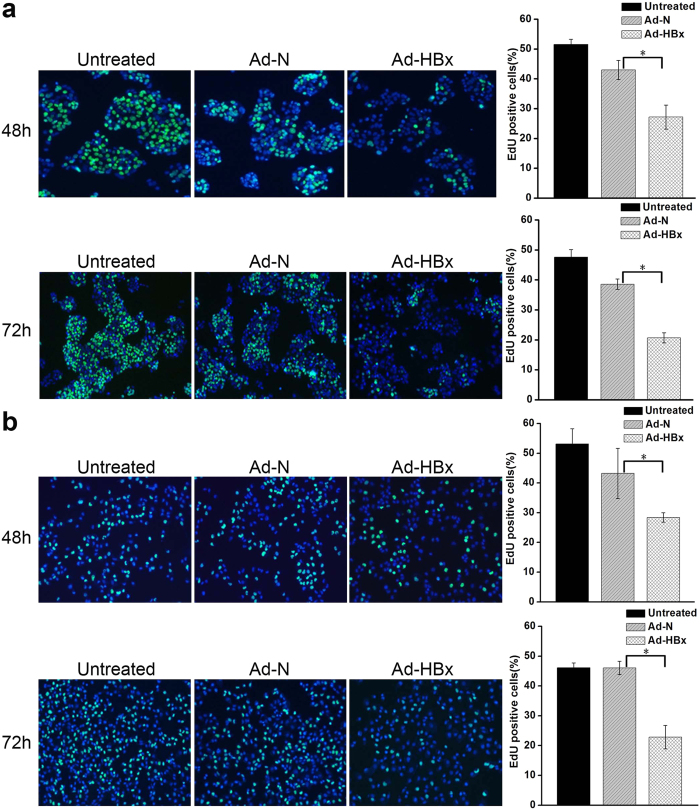
The effects of HBx on EdU incorporation into HepG2 and SK-HEP-1 cells. (**a**,**b**) Representative micrographs (left) and quantification analysis (right) of EdU-labeled cells of HepG2 and SK-HEP-1 at 48 h and 72 h post-infection. EdU-labeled cell (green) numbers of Ad-HBx-48 and Ad-HBx-72 were compared with those of Ad-N-48 and Ad-N-72, respectively. Results are representative of three independent experiments. Values represent mean ± SD. *indicates a significant difference between Ad-HBx-48 and Ad-N-48 or Ad-HBx-72 and Ad-N-72 by Student’s *t* test (*p* < 0.05).

DNA synthesis is an important part of nucleic acid metabolism^17^. To further confirm that HBx disrupted nucleic acid metabolism, we conducted an EdU incorporation assay. At 48 and 72 h after infection with Ad-HBx, EdU is an analog of thymidine, which is incorporated into DNA during DNA synthesis. EdU-labeled cells were respectively 27.17 ± 4.02% and 20.67 ± 1.66% for HepG2 (Fig. 4a), and 28.40 ± 1.58% and 22.84 ± 3.89% for SK-HEP-1 (Fig. 4b), which were significantly lower than those infected with Ad-N, or untreated cells (*p* < 0.05).

### HBx affected the expression profiles of DNA damage-related genes

**Figure 5:**
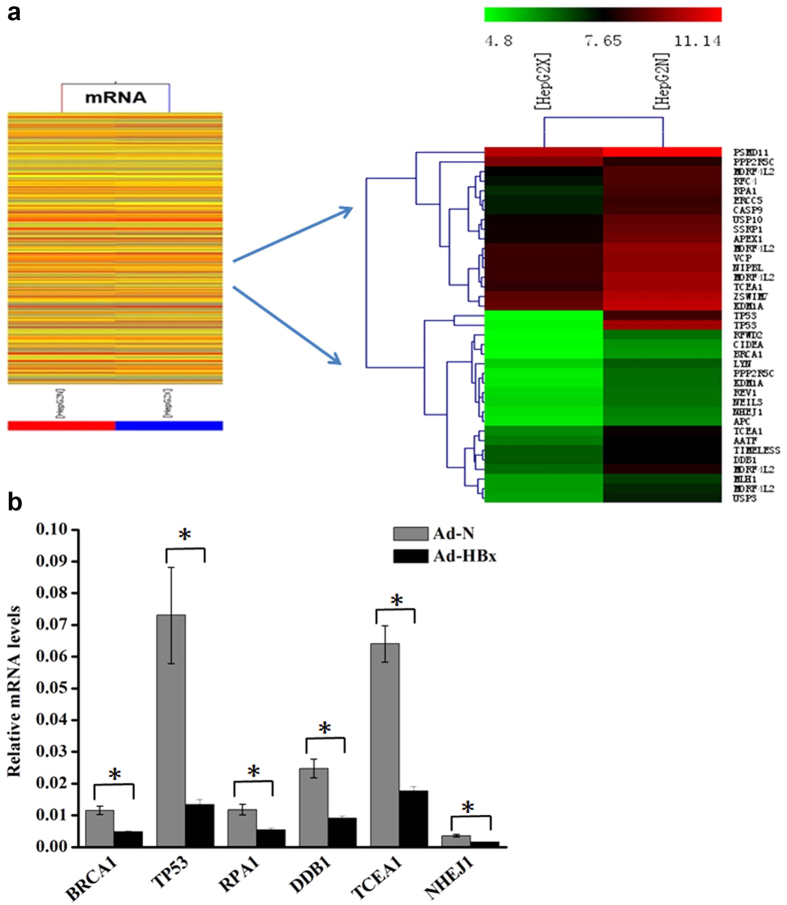
HBx affected gene expression profiles of cells. (**a**) Shows the expression profiles of mRNA (left) and DNA damage-related genes (right). Green or red on the heat map indicated a decrease or an increase in the mRNA level and color intensities correspond to relative signal levels on a logarithmic scale. (**b**) qRT-PCR validation of representative mRNA. Data were normalized to *GAPDH* and represent the mean of three experimental replicates. *indicates a significant difference between cells infected Ad-N and Ad-HBx by Student’s *t* test (*p* < 0.05).

To disclose the mechanism of HBx-associated metabolic abnormalities, we detected the differential expression profiles in HepG2 cells infected with Ad-HBx or Ad-N by mRNA microarray analysis (Fig. 5a). The mRNA microarray analysis showed that the expression levels of 966 genes were remarkably altered, 381 of those genes were upregulated (≥2-fold) and 585 genes were downregulated (≥2-fold) in cells infected with Ad-HBx. Within the altered genes, 29 were DNA damage-related genes involved in DNA damage response, nucleotide-excision repair, signal transduction in response to DNA damage, double-strand break repair, and DNA repair, and were found to be downregulated under HBx induction (Fig. 5a). To validate the results, we performed qRT-PCR for a selection of 6 differentially expressed DNA damage-related genes in HepG2 cells infected with Ad-N or Ad-HBx (Fig. 5b). The results of qRT-PCR were consistent with those of mRNA microarray analysis.

### HBx induced genomic instability

**Figure 6:**
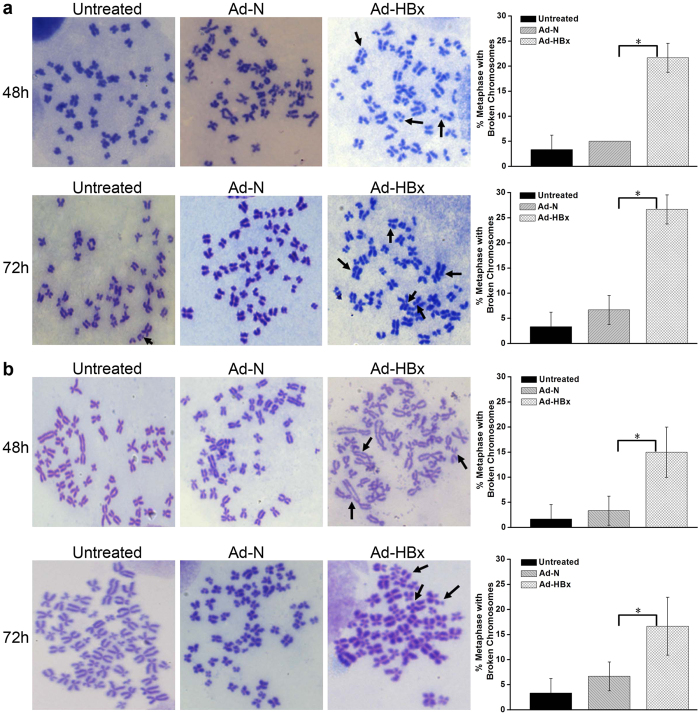
HBx induced genomic instability. (**a**,**b**) Chromosome breaks in HepG2 and SK-HEP-1 cells infected with Ad-HBx and Ad-N, or untreated were detected. Left panel: representative images of metaphase chromosomes. Arrows indicate the broken chromosomes. Right panel: quantification of metaphase spreads with broken chromosomes. Values are means ± SD from 3 independent experiments. *indicates a significant difference between Ad-HBx-48 and Ad-N-48 or Ad-HBx-72 and Ad-N-72 by Student’s *t* test (*p* < 0.05).

To examine whether HBx induced genome-wide chromosomal aberrations, we got metaphase chromosome spreads of cells infected with Ad-HBx or Ad-N, or untreated, counted 20 metaphase spreads per group, and conducted 3 independent experiments. As shown in Fig. 6, there were fewer metaphase spreads with broken chromosomes in cells infected with Ad-N and untreated, whereas in contrast, the percentage was significantly increased in cells infected with Ad-HBx (*p* < 0.05). In HepG2 and SK-HEP-1 cells infected with Ad-HBx, the average at 48 h and 72 h post-infection were respectively 21.67 ± 2.89% and 26.67 ± 2.89%, and 15.00 ± 5.00% and 16.67 ± 5.77%. The results suggested that HBx induced genomic instability.

### HBx induced DNA damage

**Figure 7:**
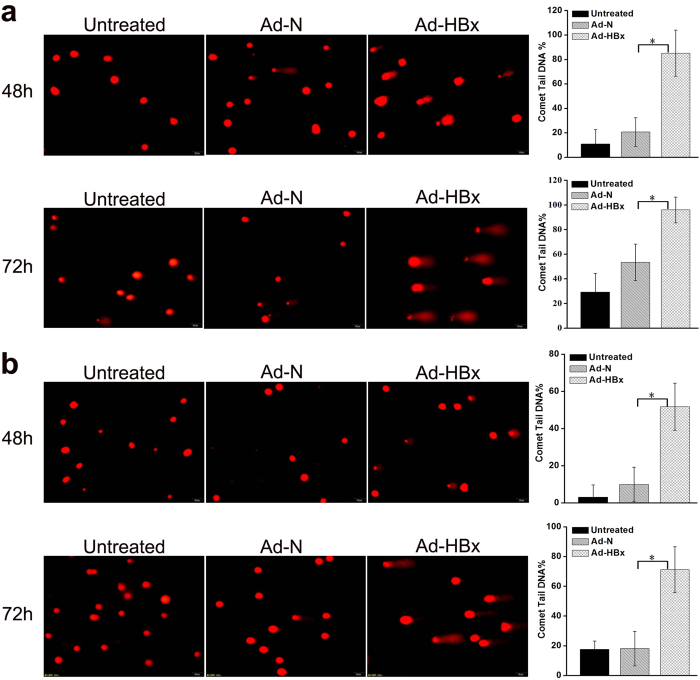
HBx-induced DNA damage was detected by comet assays. (**a**,**b**) DNA damage in HepG2 and SK-HEP-1 cells infected with Ad-HBx and Ad-N, or untreated was detected by comet assays, respectively. Left panel: representative images of comet assay (Scale bar, 50 μ m). Right panel: quantification of comet tail DNA%. Values are means ± SD from 3 independent experiments. *indicates a significant difference between Ad-HBx-48 and Ad-N-48 or Ad-HBx-72 and Ad-N-72 by Student’s t test (*p* < 0.05).

DNA damage can be evaluated by comet assay^18^,^19^, which was employed in this study to determine whether HBx could result in DNA damage. As shown in Fig. 7, at 48 h and 72 h post-infection, the nuclei from both cell lines infected with Ad-HBx showed higher damage as evidenced by increased percentage of tail DNA.

**Figure 8:**
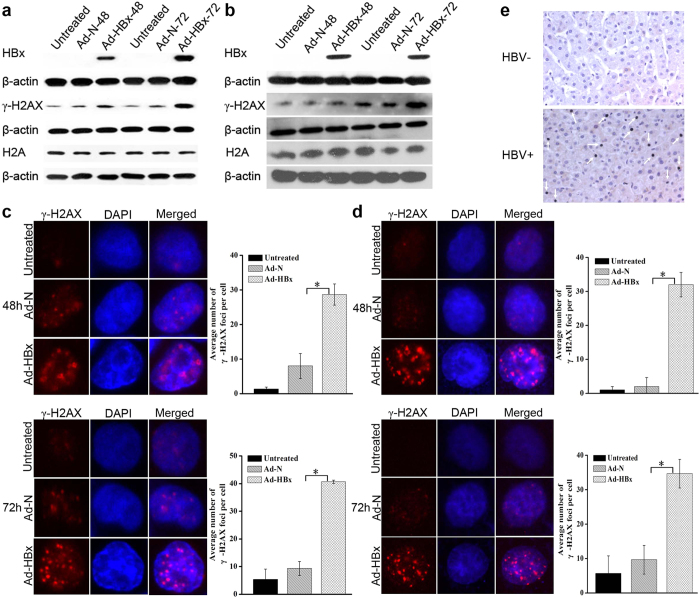
HBx-induced DNA damage was detected by Western blotting, immunofluorescence and immunohistochemistry. (**a**,**b**) Representative Western blotting of HBx, H2AX and γ-H2AX protein expression in HepG2 and SK-HEP-1 cells infected with Ad-HBx and Ad-N, or untreated. (**c**,**d**) Representative immunostaining (left) and quantification (right) of γ-H2AX in HepG2 and SK-HEP-1 cells infected by Ad-HBx or Ad-N, or untreated cells. Results are representative of three independent experiments. *p < 0.05 for comparison between Ad-HBx-48 and Ad-N-48 or Ad-HBx-72 and Ad-N-72 by Student’s *t* test. (**e**) Representative images of γ-H2AX expression in liver tissues of individuals infected with HBV by immunohistochemistry. DNA damage hepatocytes are marked with arrows.

When DNA is damaged, H2AX becomes phosphorylated at serine 139, which is then called γ-H2AX^20^. Therefore, the expression of γ-H2AX was detected by Western blotting and immunofluorescence. As shown in Fig. 8a,b, HBx induced a time-dependent increase in γ-H2AX levels, but had no effect on expression of total H2AX. Figure 8c,d showed the γ-H2AX foci induced by HBx. The average foci numbers in HepG2 and SK-HEP-1 cells infected with Ad-HBx at 48 h and 72 h post-infection were respectively 28.67 ± 3.06 and 40.67 ± 0.58, and 32 ± 3.61 and 34.67 ± 4.16, which were significantly different from those in cells infected with Ad-N. Similarly, higher levels of γ-H2AX were detected in liver tissues of individuals infected with HBV compared with those of individuals uninfected with HBV by immunohistochemistry analyses (Fig. 8e). Collectively, our results suggested that HBx induced DNA damage.

## Discussion

HBx has been reported to be associated with HBV-related hepatocarcinogenesis, thus the identification of the underlying mechanisms of HBx-mediated carcinogenesis is an important research topic. Metabolomics is an approach for rapidly identifying global metabolic changes in biological systems, and has been widely used for the diagnosis and evaluation of diverse diseases and therapies^21^. Here, an NMR-based metabolomics approach was applied to identify the distinguishing metabolites under HBx induction.

Glucose is the main source of energy and precursor for biosynthesis in cells^22^. Our results showed that the levels of glucose and its phosphorylated product, glucose-6-phosphate, were disordered after Ad-HBx infection. Meanwhile, lactate as the end product of glycolysis was also found to be abnormal in cells infected with Ad-HBx. While previous studies have reported HBV-induced glucose metabolism abnormalities^6^,^23^, to the best of our knowledge, this is the first study that reports the association of HBx and glucose metabolism abnormalities. Meanwhile, levels of a variety of amino acids such as glutamate, glutamine, creatine, alanine, threonine, glutamate, serine and glycine, were disordered after Ad-HBx infection. These distinguishing metabolites were involved in amino acid metabolism, suggesting dysfunction of amino acid metabolism in cells infected with Ad-HBx. These results are in agreement with the amino acid metabolism abnormalities of HCC and liver cirrhosis^5^,^10^.

We did not detect lipids in the current study, because only water-soluble substances were extracted in the research investigating metabolic profiles of cells by an NMR-based metabolomics approach. However, we found that the levels of N-acetylaspartate (NAA) and choline were significantly different in cells infected with Ad-N *versus* Ad-HBx. NAA is involved in fatty acid metabolism^24^, while choline is a precursor of phosphatidylcholine and has a key role in systemic lipid metabolism^25^,^26^. Therefore, the abnormal levels of NAA and choline under HBx induction indicated that HBx might also disrupt lipid metabolism. Our result was consistent with previous studies that show that HBx resulted in lipid accumulation and activated lipogenic genes^7^,^13^. Combined, these findings indicated that HBx could disturb lipid metabolism.

Intriguingly, we found that in cells infected with Ad-HBx, uridine, guanosine and inosine had high VIP scores and showed a significant decrease in concentration (*p* < 0.05). Meanwhile, uracil and xanthine also were important in providing class separation. Their concentration from ^1^H-NMR was relatively low; however, they had high VIP scores, which piqued our attention. Uridine, guanosine, inosine, uracil and xanthine as important metabolic intermediates of nucleic acid metabolism, are involved in the biosynthesis of DNA and RNA. The EdU incorporation assay showed that HBx inhibited DNA synthesis. Overall, the results suggested that HBx could induce abnormalities of nucleic acid metabolism. A recent study showed that adipocyte DNA damage could aggravate metabolic abnormalities, and the abnormalities could be ameliorated by reduction of DNA damage^27^. As is well known, DNA damage affects all DNA metabolic processes, for example, DNA replication and transcription^28^. Pal *et al*. reported that amelioration of DNA damage by resveratrol restored protein and nucleic acid metabolism in the brain^29^. Thus, whether the metabolic abnormalities are related to DNA damage remains to be clarified in future studies.

Our previous study showed that HBx was able to induce G2/M phase arrest in HCC cells^30^. G2/M check-point has an important role in the replication of genome. When DNA lesions arise, mitosis is prevented to minimize the detrimental effects and provide an opportunity to repair these genomic lesions^31^. So, we speculated that HBx could induce DNA damage. In the current study, gene-expression profiles showed that 29 genes involved in DNA damage response, nucleotide-excision repair, signal transduction in response to DNA damage, double-strand break repair, and DNA repair, were differentially expressed. Analyzing the chromosomal aberrations in the whole genome showed that HBx induced genomic instability. In addition, HBx-induced DNA damage was further demonstrated by comet assay, Western blotting, immunofluorescence, and immunohistochemistry. Numerous studies have reported that DNA damage plays an important role in carcinogenesis^32^,^33^. Under normal physiological conditions, eukaryotes can repair damaged DNA through the signal pathways of DNA damage response^34^. Our study, using mRNA microarray analysis, suggested that genes of HBx-induced DNA damage response and DNA repair were downregulated. The decreased gene expression might be due to the abnormalities of nucleic acid synthesis.

M Strubin *et al*. reported that HBx interfered with cell cycle and promoted HBV gene expression by interacting with DNA damage binding protein 1 (DDB1)^35^,^36^, and the cytotoxic activities of HBx were maintained when forming a complex with DDB1 in the nucleus^37^. In our study, we also confirmed that HBx colocalized with DDB1 mainly in nucleus in cells infected with Ad-HBx (Supplementary Figure S1). DDB1 is a subunit of an E3 ubiquitin ligase complex, which can recognize DNA lesions and plays an important role in DNA repair^35^. HBx has the binding site of DDB1^38^. So, we speculate that HBx-induced DNA damage may be due to its interaction with DDB1, which disrupts the DNA repair activities of DDB1.

In conclusion, we speculate that HBx first induces DNA damage, and then disrupts nucleic acid metabolism. The metabolism abnormalities block DNA repair and induce the occurrence of HCC. However, further investigations are needed to study how HBx-induced DNA damage disrupts nucleic acid metabolism.

## Materials and Methods

### Chemicals and reagents

Dulbecco’s modified Eagle medium (DMEM) was purchased from Gibco (Carlsbad, CA). Fetal bovine serum (FBS) was purchased from Biowest. ViraPower adenovirus expression system and Gateway system were obtained from Invitrogen (Carlsbad, CA). Cell-Light^TM^ EdU kit was purchased from Guangzhou RiboBio Co., Ltd. (Guangzhou, China). 2,2-dimethyl-2-silapentane-5-sulfonate (DSS) standard solution was certified by Anachro (ACDSS). RIPA buffer, PMSF and Enhanced BCA Protein Assay Kit were purchased from Beyotime biotechnology, China.

### Cell lines and cell culture

Hepatocellular carcinoma cell lines (HepG2 and SK-HEP-1) and a human embryonic kidney cell line (293A) were obtained from ATCC and maintained in DMEM supplemented with 10% Fetal Bovine Serum (FBS) in a humidified atmosphere with 5% CO_2_ at 37 °C.

### Generation of recombinant adenovirus

HBx expressing recombinant adenovirus (Ad-HBx) was prepared as previously described^30^. Briefly, cDNA coding HBx was cloned into pENTR11 and then the plasmid expressing HBx (pENTR11-HBx) was recombined with pAd using Gateway system (Invitrogen). Ad-N as a control adenovirus did not express a foreign gene. Virus was produced in 293A cells and purified by cesium chloride gradient centrifugation. HBx in our study was from Hepatitis B virus genome, subtype *ayr*, and its sequence was shown in supplementary data.

### Metabolite extraction

Cells were seeded in 6 cm dishes and grew to 80%~90% confluence, then were infected with Ad-HBx or Ad-N at an MOI of 20 in DMEM supplemented with 2% FBS. After 2 h, the cells in each dish were detached using trypsin/EDTA solution and cultured in three dishes for 48 h or 72 h. At 48 and 72 h post-infection, the cells were harvested, and rapidly quenched by liquid nitrogen and stored at −80 °C. After storage at −80 °C for several days, the cells were lyophilized in a vacuum freezing dryer. More than 50 mg lyophilized cells per sample were weighed and resuspended in 1 ml milliQ water followed by vortexing for 1 min. After sonication, the samples were centrifuged at 13000 rpm at 4 °C for 10 min, and the supernatant was filtered using 3-kDa microcentrifuge filters, and 450 μL filtrate per sample was transferred into a clean microfuge tube containing 50 μL ACDSS (4.136 mM) as an internal standard at a chemical shift of 0.0 ppm.

### ^1^H-NMR spectroscopy

All NMR experiments were performed on Bruker AV III 600 MHz spectrometer (Bruker Biospin, Milton, Canada) equipped with an inverse cryoprobe operating at 600.13 MHz. All NMR spectra of samples were acquired using a standard Bruker noesygppr1d pulse sequence, and a total of 256 scans were collected into 32768 data points over a spectral width of 8000.00 Hz.

### NMR data analysis

Identification and quantification of individual metabolites were performed using Chenomx NMR Suite software (version 7.7, Chenomx, Edmonton, Canada). ^1^H-NMR spectra were compared against Chenomx library that contained the unique ^1^H-NMR spectra of each standard compound quantified by a known reference signal (DSS). Comparisons of NMR spectra with this database produced a list of compounds and their concentration, and the absolute concentration of each compound was normalized based on the weight of samples. A supervised partial least squares-discriminant analysis (PLS-DA) approach was chosen to compare the variance of metabolite concentration between Ad-HBx-48 and Ad-N-48 or Ad-HBx-72 and Ad-N-72. Student’s *t* test was used to compare between groups for the discriminant variables obtained from PLS-DA and a *p* value < 0.05 was considered significant.

### EdU incorporation assay

To further study the effect of HBx on nucleic acid metabolism, we analyzed DNA synthesis using 5-ethynyl-20-deoxyuridine (EdU) incorporation assay. HepG2 and SK-HEP-1 cells infected with Ad-HBx or Ad-N, or untreated were seeded in 96 well plates and allowed to grow for 46 h or 70 h, then labeled by incubation with 50 μM EdU for 2 h. After fixation, cells were stained with Apollo® fluorescent dye, Apollo^®^488, then counterstained with Hoechst33342. Labeling of proliferating cells was imaged by fluorescence microscopy.

### Gene expression profiles

Total RNA was isolated using Trizol reagent (Invitrogen) from HepG2 cells infected with Ad-N or Ad-HBx according to the manufacturer’s instructions. RNA was reverse transcribed with Cy3-labelled-CTP and fluorescence quantified using NanoDrop ND-1000 (Thermo Scientific). After hybridization, GeneChips were scanned by Agilent Microarray Scanner (Agilent p/n G2565BA).

### Quantitative real time PCR (qRT-PCR)

Total RNA was isolated with Trizol from HepG2 cells infected with Ad-N or Ad-HBx according to the manufacturer’s instructions and was then reverse transcribed with a cDNA Synthesis Kit (TaKaRa). The resulting cDNA was used for qRT-PCR analysis using SYBR Green (TaKaRa) with gene-specific primers, and data were normalized to glyceraldehyde-3-phosphate dehydrogenase (GAPDH). The qRT-PCR primers were shown in Table 2.

### Analysis of chromosomal aberrations

HepG2 and SK-HEP-1 cells infected with Ad-HBx or Ad-N, or untreated were treated with 0.4 μg/ml colcemid for 4 h at 44 or 68 h post-infection, trypsinized and harvested. After hypotonic treatment with 0.075 M KCl for 10 min at 37 °C, cells were fixed with 3:1 mixture of methanol:acetic acid thrice. Then, cells were dropped on precooled glass slides to obtain metaphase chromosome spreads. Chromosomes were stained with Giemsa and imaged by a microscope with a 100X objective lens.

Comet assay

Comet assay was performed as previously described^39^,^40^. Briefly, HepG2 and SK-HEP-1 cells were infected with Ad-HBx or Ad-N, or untreated. At 48 and 72 h post-infection, the cells were collected and resuspended in PBS, and 10 μl single-cell suspensions was mixed with 75 μl 0.7% low-melting point agarose, layered onto a glass microscope slide and left in ice-cold lysis buffer (1 M NaCl, 1 mM EDTA, 10 mM Tris-HCl, 30 mM NaOH, 1% Triton X-100 and 10% DMSO, pH 10.0) for 2 h. All slides were placed in alkaline electrophoresis buffer (300 mM NaOH and 1 mM EDTA, pH 10.0) for 40 min, and electrophoresis was performed at 25 V for 20 min. Then the slides were neutralized with buffer (0.4 M Tris-HCl, pH 7.5) thrice, stained with propidium iodide (2.5 μg/mL) for 10 min and imaged by a fluorescence microscope.

### Western blotting

Western blotting was performed as previously described^41^,^42^. Briefly, HepG2 and SK-HEP-1 cells were infected with Ad-HBx or Ad-N, or untreated. At 48 and 72 h post-infection, the cells were collected, washed in PBS, and lysed in RIPA buffer containing 1 mM PMSF. Total protein concentration was quantified with the Enhanced BCA Protein Assay Kit. The cell lysates were separated by SDS-PAGE and transferred to polyvinylidene fluoride (PVDF) membranes. The membranes were blocked in Tris-buffered saline containing 0.1% Tween-20 (TBS-T) and 5% nonfat milk for 2 h at room temperature, and incubated with primary antibodies against HBx, γ-H2AX (phosphorylated on Ser139), H2AX and β-actin overnight at 4 °C, followed by incubation with horseradish peroxidase-conjugated secondary antibodies at 37 °C for 1h. The blots were detected using the enhanced chemiluminescence system (Millipore).

### Immunofluorescence

Cells infected with Ad-HBx or Ad-N, or untreated were fixed with 4% paraformaldehyde in PBS for 20 min, then permeabilized with 0.5%Triton X-100 in PBS for 15 min and blocked with 5% goat serum for 30 min at room temperature as specified in a previous report^43^. For detection of the γ-H2AX expression, the fixed cells were incubated with anti-γ-H2AX antibody overnight at 4 °C followed by incubation with TRITC-conjugated goat anti-rabbit IgG for 1 h at 37 °C, and stained with DAPI to visualize the cell nucleus. The images were obtained by using a fluorescence microscope.

### Immunohistochemistry

Human tissue arrays were provided by Shanghai Biochip Co., Ltd. (Shanghai, China) including liver tissues from 20 patients with or without HBV infection. DNA damage was evaluated by immunohistochemistry using anti-γ-H2AX antibody, and the ZSGB-BIO SPlink Detection Kit (Beijing, China). After standard dewaxing, washing, neutralization of endogenous peroxidase and heat-induced antigen-retrieval, tissues were blocked with goat serum for 15 min at room temperature and then incubated with anti-γ-H2AX antibody overnight at 4 °C followed by incubation with biotin-conjugated goat anti-rabbit IgG for 15 min at 37 °C . Subsequently, the sections were treated with horseradish peroxidase-conjugated streptavidin, visualized by incubation with 3, 3′-diaminobenzidine (DAB) and counterstained with hematoxylin.

### Statistical analysis

Results are presented as mean ± standard deviation (SD) of the data from three independent experiments. For statistical comparison between groups, Student’s *t* test was used, with a *p* value less than 0.05 considered significant.

## Additional Information

**How to cite this article**: Yue, D. *et al*. Hepatitis B virus X protein (HBx)- induced abnormalities of nucleic acid metabolism revealed by ^1^H-NMR-based metabonomics. *Sci. Rep.* **6**, 24430; doi: 10.1038/srep24430 (2016).

## Figures and Tables

**Table 1:** Summary of all identified and analyzed metabolites in HepG2 cell extracts and their numbers on ^1^H-NMR spectra.

Metabolite	No.	Metabolite	No.	Metabolite	No.
2-Oxoglutarate	1	Glutamate	21	Pantothenate	41
Acetate	2	Glutamine	22	Phenylalanine	42
Alanine	3	sn-Glycero-3-phosphocholine	23	Proline	43
Arginine	4	Glycine	24	Putrescine	44
Asparagine	5	Guanosine	25	Pyroglutamate	45
Aspartate	6	Histidine	26	Serine	46
Betaine	7	Hypoxanthine	27	Succinate	47
Choline	8	Inosine	28	Taurine	48
Creatine	9	Isoleucine	29	Threonine	49
Cystathionine	10	Lactate	30	Thymine	50
Cysteine	11	Leucine	31	Tryptophan	51
Cystine	12	Lysine	32	Tyrosine	52
UDP-N-GlcNAc	13	Malate	33	Uracil	53
Dimethylamine	14	Mannose	34	Uridine	54
Ethanol	15	Methanol	35	Valine	55
Ethanolamine	16	Methionine	36	Xanthine	56
Formate	17	N-Acetylaspartate	37	Acetone	57
Fumarate	18	NAD^+^	38	Sarcosine	58
Glucose	19	Nicotinurate	39	Glucose-6-phosphate	
β-Alanine	20	O-Phosphocholine	40	Oxypurinol	

UDP-N-GlcNAc: UDP-N-Acetylglucosamine.

**Table 2:** The primer nucleotide sequences and annealing temperatures of quantitative real time PCR.

Gene	Primer nucleotide sequences	Annealing temperature(°C)
GAPDH	F: 5′-GGGAAACTGTGGCGTGAT-3′R: 5′-GAGTGGGTGTCGCTGTTGA-3′	60
BRCA1	F: 5′-CACCAAGGTCCAAAGCGA-3′R: 5′-TTGCATGGAAGCCATTGTC-3′	60
TP53	F: 5′-GCCCATATCTGTGAAATGCT-3′R: 5′-ACTTGACAACTCCCTCTACCTAAC-3′	60
RPA1	F: 5′-TCAAGCACTATCATTGCGAATC-3′R: 5′-TTGTCCTTCTGCGTCAAACC-3′	60
DDB1	F: 5′-TCAAAAGGATAGCGCTGCCA-3′R: 5′-GTCCAGCAGGAGGTTGTACC-3′	60
TCEA1	F: 5′-CTTCTGATTCTGTGCGGTTGA-3′R: 5′-ACTAGCCATTTCCTCTGCTGTC-3′	60
NHEJ1	F: 5′-GAAGGACGCTGCTCACCCTA-3′R: 5′-ATGCCCATCAGAGGACGAAT-3′	60
